# From brain to spinal cord: neuromodulation by direct current stimulation and its promising effects as a treatment option for restless legs syndrome

**DOI:** 10.3389/fneur.2024.1278200

**Published:** 2024-01-25

**Authors:** Christina A. H. Dirks, Cornelius G. Bachmann

**Affiliations:** SomnoDiagnostics, Osnabrueck, Germany

**Keywords:** neuromodulation, transcranial direct current stimulation, transcutaneous spinal direct current stimulation, restless legs syndrome, chronic pain

## Abstract

Neuromodulation is a fast-growing field of mostly non-invasive therapies, which includes spinal cord stimulation (SCS), transcranial direct current stimulation (tDCS), vagal nerve stimulation (VNS), peripheral nerve stimulation, transcranial magnetic stimulation (TMS) and transcutaneous spinal direct current stimulation (tsDCS). This narrative review offers an overview of the therapy options, especially of tDCS and tsDCS for chronic pain and spinal cord injury. Finally, we discuss the potential of tsDCS in Restless Legs Syndrome as a promising non-invasive, alternative therapy to medication therapy.

## Introduction

Neuromodulation by transcranial direct current stimulation (tDCS) as a non-invasive method for modulation of excitability of the human cortex (1) first came to attention of physicians and researchers in the early 1960s as a therapy option for chronic, therapy-resistant pain (2). During this time, first animal studies in rats and cats were carried out (3, 4). Shortly thereafter, attempts to reduce pain using electronic deep brain stimulation in humans began (5). Shaely et al. (6) were the first who presented a case report of a patient in whom improvements of intractable pain were achieved through spinal cord stimulation (SCS) using electrical impulses. These were the beginnings of a new direction in medical pain treatment: away from invasive therapy methods to relieve pain such as cutting nerves, towards reversible, modulatory treatment options (7). However, for tDCS it was not until the turn of the millennium to become firmly established after the plasticity-generating effects of this method in the human brain could be demonstrated using transcranial magnetic stimulation (TMS) (1, 8).

Currently, neuromodulation is a fast-growing field of mostly non-invasive therapies, which includes, in addition to SCS and tDCS, vagus nerve stimulation (VNS), peripheral nerve stimulation, transcranial magnetic stimulation (TMS) and transcutaneous spinal direct current stimulation (tsDCS) (9). The latter is a non-invasive method – like tDCS—using constant currents through electrodes which are directly applied to the skin via a dermal patch instead of applying a pulse stimulation through epidural electrodes as is it the case in SCS (10). Therefore, tsDCS became a non-invasive and inexpensive alternative to SCS to modulate spinal cord functions (11–13).

Both, tDCS and tsDCS are methods using low-level electrical currents (< 1–2 mA) via anodal or cathodal stimulation to trigger a polarization effect (14–16). Thus, the neuromodulating potential of these methods lies in the change of neuronal excitability (17) instead of magnetic fields as in TMS. In sum, tDCS – like TMS – now has a wide range of clinical indications.

Transcranial anodal stimulation leads to a short-term increase of cortical excitability by increasing the discharge rate of active neurons through hyperpolarizing dendrites and depolarizing the cell body. On the other hand, cathodal stimulation leads to an inhibition of the neuronal functions (1, 18–20). Interestingly, with tcDCS it is exactly the other way around. Moreover, tDCS induced long-term effects may be due to a subthreshold change in the neural resting membrane potential by up-or downregulation of membrane receptors that lead to changes in cortical synapse strength (3, 20–25). Liebetanz et al. (26) provided evidence that modification of N-methyl-D-aspartate (NMDA) receptors is critical for tDCS induced long-term potentiation and long-term depression. Monte-Silva et al. (27) analyzed the effects of transcranial anodal DCS on neuronal plasticity of the human motor cortex by repeated stimulation. The results showed that repeated stimulation three and 20 min after the first stimulation phase had the strongest effect on cortical excitability, which lasted up to 24 h. Thus, repeated tDCS can induce long-term potentiation in the motor cortex (27). Early on, Paulus et al. (25) postulated that tDCS exerts its effects not only locally, but also to cortical network level (28, 29). In line with this, currently, mounting evidence challenges the traditional view of increasing excitability by anodal and decreasing excitability by cathodal stimulation. In a study of the connectivity of motor-networks in the motor-cortex (M1 areal) and the cerebellum Calzonari et al. (18) provided a more complex picture at network level. With the growing understanding of how tDCS works, possible applications are also increasing. Clinical application of repeated tDCS currently reveals promising therapeutic approaches in many different medical areas, e.g., chronic pain, spinal cord injuries and restless legs syndrome (30, 31).

## tDCS and tsDCS as a neuroplasticity-inducing stimulation method in the treatment of chronic pain and spinal cord injury

### Chronic pain

In the field of chronic pain, the need for new, effective treatment options is particularly high, since many pain patients have exhausted the standard treatment methods without having achieved a significant improvement in their symptoms. It is postulated that the analgesic effect is achieved through the property of tDCS to influence neuronal activity by polarizing the resting membrane (9, 32). Clinical applications of tDCS were investigated in randomized controlled studies, such as the treatment of migraine patients. The results revealed that in chronic pain, both, repetitive TMS and tDCS, can achieve similar, albeit transient, improvements after about two weeks of stimulation that were about 30% to 60% improvement on a visual analog scale (33). Nitsche and Paulus (32) were among the first to study the use of tDCS in chronic pain patients more than 20 years ago. They could show that tDCS is able to induce sustained cortical excitability elevations. Moreover, they provide evidence for the feasibility of inducing long-lasting motor cortical excitability, which increased approximately 150% above baseline for up to 90 min after the end of stimulation. The results were comparable with the effects of TMS in this field (32). Another study by Fregni et al. (34) investigated the effects of anodal tDCS on subjective perception of pain in chronic pain patients. For this purpose, primary motor cortex (M1) was stimulated with 2 mA for 20 min on five consecutive days. Pain sensation was measured with a visual analogue scale. Significant differences from the initial measurement were found after the third day of tDCS. A gradual reduction in subjective pain perception was also evident on the fourth and fifth day, with the greatest effect on the fifth day. Interestingly, pain reduction was still clearly visible in follow-up studies after 14 days (34).

The potential option of chronic pain treatment by direct current stimulation, which was applied transcutaneously to spinal regions, was also examined in this context. Cogiamanian et al. (35) for example could provide evidence, that the spinal flexor reflex in healthy subjects is reduced by 40% immediately after a 15-min tsDCS stimulation interval and by 47% after a 30-min tsDCS stimulation interval. They concluded that tsDCS has the potential to induce even long-lasting changes in the central pain pathways in human beings. Other studies by Meyer-Frießem et al. (36) and Perrotta et al. (37) in healthy subjects provided further evidence that pain sensitivity can be suppressed up to 60 min by anodal tsDCS and suggested that tsDCS may provide an effective, non-invasive tool in pain management.

### Spinal cord injury

There are only a few studies investigating the effectiveness of tDCS in spinal cord injury. In this field, TMS appears to be superior to DCS when applied transcranially (9). However, there is increasing evidence that the therapeutic efficacy of DCS becomes more promising when the method is applied transcutaneously in the affected region of the spinal cord. Anodal transcutaneous spinal direct current stimulation (tsDCS) normalizes reflex hyperexcitability in patients with lesions in the upper motor neurons by decreasing spinal reflex excitability and influencing the ascending and descending spinal pathways (34). Moreover, there is mounting evidence that it can also induce prolonged neuroplasticity changes in the investigated function (38–41).

Like tDCS, tsDCS works by altering the membrane potential of neurons by direct current, but the technique does not trigger neuron action potentials, as TMS does. However, according to Hebbian’s law of neuroplasticity: “neurons that fire together wire together,” neuron action potentials are needed for spinal circuits to reform (42, 43). For that purpose, many studies in this area have combined the use of tsDCS with motor tasks. For example, different studies revealed the enhanced positive therapeutic effects of tsDCS in patients after spinal cord injury and considerable walking impairment on walking rehabilitation, dynamic balance control and locomotor-training if applied in combination (44, 45).

In a study with healthy subjects Albuquerque et al. (46) combined tsDCS with a 20 min treadmill exercise immediately, 30 min and 60 min after stimulation. They provided evidence that anodal tsDCS led to a significant decrease in Hoffmann reflex (Hmax/Mmax-ratio) and nociceptive flexion reflex immediately and 30 min after anodal current stimulation. Furthermore, the nociceptive flexion reflex was significantly increased after cathodal stimulation, whereas cathodal stimulation had no effect on Hmax/Mmax-ratio in this study (47).

Even if the database in this field is still in need for more studies, it can be summed up that tsDCS seems to result in reliable improvements of pathophysiological impairments of spinal cord function, particularly when applied in combination with relevant motor tasks. Thus, tsDCS may have the potential for neuromodulation in spinal cord-injured subjects (38).

### The potential of tsDCS as a new non-drug therapy in restless-legs-syndrome: current state of research

Restless Legs Syndrome (RLS) is a movement disorder and one of the most common neurological diseases in western populations with a prevalence of 5%–10% (46, 48). It is characterized by an imperative urge to move the legs combined with somatosensory abnormal sensations or pain and can also affect—more rarely—the arms, bladder, genital and rectal regions. The symptoms, which occur in the circadian rhythm with a focus on evening and night hours, intensify in phases of rest and relaxation. On the other hand, with movement they improve or suspend. The pathophysiology of RLS is likely heterogeneous and not fully understood (46, 49). Studies using transcranial magnetic stimulation (TMS) to investigate motor cortex excitability in RLS-patients provide abnormal fluctuations in this area and a reduced intracortical inhibition (50–55). Moreover, several pathophysiological mechanisms may be involved, including low ferritin, glutamate, adenosine, creatinine, high level of urea in the blood and a lack of vitamin B12 and B6 (46, 56).

Currently, the most important therapeutic intervention is still pharmacotherapy with drugs mostly based on dopamine and its agonists (57). Therefore, most disease models trying to clarify the pathophysiological mechanisms of RLS focus on changes in dopamine neurotransmission and connectivity pathways in the brain (58–62). The use of these drugs is currently viewed critically, since in many cases long-term use leads to an augmentation of the symptoms under dopamine-based drugs, and the development of effective non-drug therapy methods is therefore urgently needed.

Several studies have shown evidence that the spinal cord may be substantially involved in RLS pathophysiology (46, 63). A current focus of RLS research is therefore the possibility to change neuronal excitability in the spinal cord networks with tsDCS to relieve RLS-symptoms. This is even more important given the particularly severe side effects of dopamine-based drugs.

A study by Heide et al. (64) showed evidence that tsDCS may offer a non-invasive, painless alternative to drug treatment for RLS. In this study, promising effects of tsDCS on clinical symptoms and corresponding on spinal excitability in RLS patients were demonstrated for the first time. tsDCS was applied anodal and cathodal with 2,5 mA (and sham condition) once for 15 min at two different sessions with a resting interval of one week to avoid after-effects.

After-effects were first described for tDCS by Monte-Silva et al. (27). The authors were able to show that periodical anodal tDCS induce long-lasting, late longterm potentiation like (l-LTP) excitability enhancements of the primary motor cortex dependent on the duration of the interval between tDCS applications, because tDCS induces long-term effects via the manipulation of NMDA receptors and this effect remains present for a certain time period after stimulation (after-effect). Monte-Silva et al. provided evidence that if the second stimulation was performed during the after-effects of the first one, the combined after-effects were present for more than 24 h after tDCS, with an initially reduced, but then relevantly prolonged excitability enhancement. Furthermore, they showed that excitability enhancement could be blocked by an NMDA receptor antagonist.

### Theories about the pathogenesis of RLS from brain to spinal cord and how to link them

**Figure 1:**
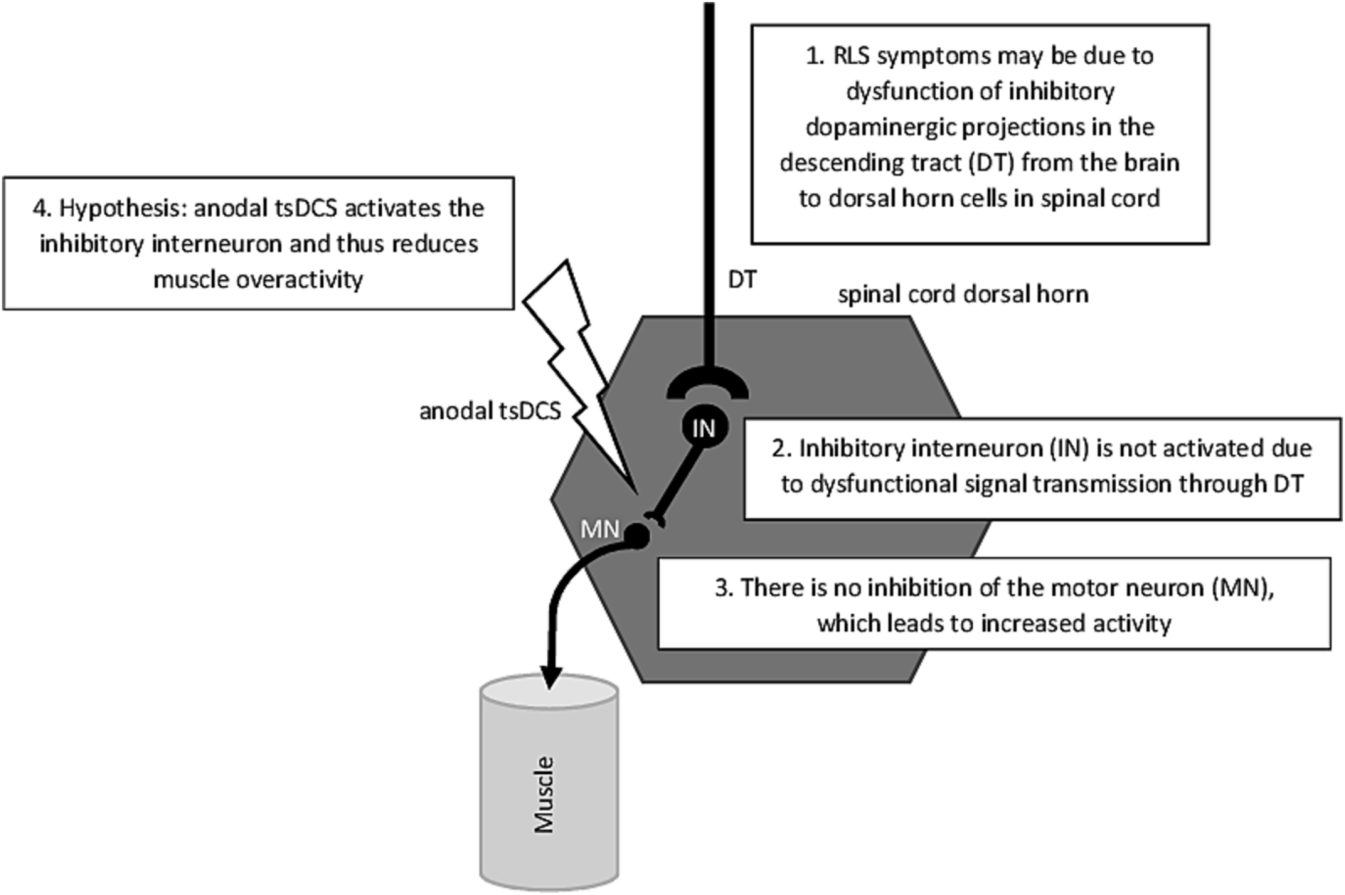
Schematic overview of the proposed effect of anodal tsDCS on RLS in the spinal network.

Heide et al. (64) provided first evidence that anodal tsDCS resulted in a significant reduction in the H2/H1-ratio of the Hoffmann-(H)-reflex, indicating a decreased excitability of the spinal cord (65–67). Typically, RLS patients show increased H2/H1-ratios during their symptomatic phase in the evening which is assumed to be caused by a compromised supraspinal inhibitory pathway projecting onto spinal motoneurons or on altered excitability in local spinal circuits (62) (see Figure 1). Cathodal and sham stimulation had no effects on H2/H1-ratios. Regarding RLS severity, measured with a visual analog scale, both anodal and cathodal tsDCS resulted in significant improvements in RLS severity, with anodal stimulation having stronger effects. Interestingly, only in the anodal stimulation condition, the decrease in RLS symptom severity was associated with a measurable reduction in the H2/H1 ratio.

The results of the study by Heide et al. (64) are consistent with the current theory of the mechanisms of neuroplasticity induced by tsDCS: In animal studies, Ahmed (68) were able to show that cathodal tsDCS leads to an increase in glutamate release and, at the same time, to a blocking of the GABA receptors which results in the stimulating effect of cathodal tsDCS. Like mentioned above, RLS patients show increased H1/H2-ratios in the symptomatic phase, which—according to this theory – could be reduced by anodal stimulation leading to a suppression of ascending spinal pathways tracts (35, 69–73).

However, as the scope of the study by Heide et al. (64) was not to explore long-term effects of stimulation, objective results were only recorded for short-term effects of tsDCS on RLS-symptoms. Patients reported that the positive or after-effects of tsDCS lasted for a few more hours after stimulation, but this information was not quantitatively gathered using a validated scale (64). These results have been replicated by other independent research studies (73, 74).

Figure 1 gives a schematic overview of the possible mechanism that trigger RLS on spinal cord level. But what causes the dysfunction of dopaminergic projections in the descending tract described in Figure 1? To answer this question, the path leads up from the spinal cord to the brain. A well-known symptom of RLS are low brain iron levels even when RLS-patients have normal serum ferritin and no indication of peripheral iron deficiency. Rizzo et al. (75) were the first to reveal iron deficiency *in vivo* by using MRI techniques. Brain structures which show iron deficiency were above all the substantia nigra, and to a lesser degree in the putamen, caudate and the thalamus. Allen et al. (76) explained the low iron level in these brain structures by a lack of iron crossing the blood–brain barrier resulting in a deficit of iron in critical neuronal cells of the mentioned brain structures. The authors concluded thus—providing that oxygen-transport depends on iron—a decreased level of iron in the brain should signal hypoxia. Benediktsdottir et al. (77) provided evidence that as a direct consequence of the activation of hypoxic pathways would be an increase of dopaminergic activity like it is the case in RLS patients. As Vlasie et al. (78) mentioned, the key to understand the fact why treating RLS patients with levodopa is still one of the most effective treatment strategies, is the circadian rhythm of both: dopaminergic activity and RLS symptoms with an increase in the morning and decreasing in the evening and night. Permanently increased dopamine levels in the morning and daytime result in downregulation at both the dopamine receptor and intracellular level and therefore the post-synaptic response in RLS patients is although adequate for daytime but exaggerated for nighttime, resulting in an evening and nighttime dopaminergic deficit (78) (see Figure 1).

A further consequence of the reduced brain iron levels is a lack of myelin sheaths in the brain as the synthesis also dependent on iron. Conner et al. (79) revealed significant decreases in white matter in the corpus collosum, anterior cingulate and precentral gyrus. In line with this results are the latest findings of a DTI-study by Park and colleague (61) which provide evidence of a decreased segregation in the global brain network of the RLS patients even in correlation with RLS severity. Furthermore, they found changes in local structural connectivity in regions involved in sensorimotor function, including the middle frontal gyrus, superior frontal gyrus, orbital frontal gyrus, postcentral gyrus, supplementary motor area, and thalamic substructures (pulvinar and anterior thalamic nucleus). The results provide further evidence that an altered sensorimotor network may play a pivotal role in the pathophysiology of RLS (61).

The current findings of brain abnormalities in RLS patients should not obscure the fact that there are a lot of pathophysiological theories that deal with the causes at the spinal cord level, which is also part of the CNS, and that the call for more neuroimaging approaches dealing with the structure and the function of the spinal cord in RLS is getting louder (62).

To our knowledge, there are currently two studies that attempt to close this gap. The results of those studies by Wang et al. (74) and Zeng et al. (80) not only provided a significant decrease in RLS symptoms by anodal tsDCS treatment, which lasted up to two weeks. Moreover, their studies provided first evidence by fMRI that repetitive anodal tsDCS even may have a modulating effect in the functional connectivity and gray matter volume in brain regions like visual (V1 area) and motor area networks (supplementary motor area, SMA) which correlated with the decrease in RLS symptoms assessed by the International RLS Rating Scale (74, 80). While the involvement of SMA was consistent with the hypotheses, the significant activation of the V1 area, which is important for visual information processing, might be more unexpected. The authors mentioned that in a previous study, which investigated the effects of acupuncture, activation of the occipital cortex indicated an antinociceptive effect by activating the descending inhibitory pathway (80) and that the activation of V1 area might serve as a biomarker of treatment response in the future (81) (for an overview of the studies see Table 1).

**Table 1:** Overview of the tsDCS studies in RLS and summary of the results.

Reference	Title	Type of trial	Sample size	Methods applied	Results
Heide et al. (64)	Effects of transcutaneous spinal direct current stimulation in idiopathic restless legs patients.	Double-blinded, placebo-controlled	20 patients with RLS and 14 healthy subjects	Cathodal, anodal and sham tsDCS for 15 min. With 2.5 mA. RLS symptoms were assessed by a visual analogue scale (VAS).	Anodal stimulation: ↓ H2/H1-ratio. Sham stimulation: no effects. Anodal and cathodal stimulation: ↓ VAS-scores. Sham stimulation: no effect.
Wang et al. (74)	Altered grey matter volume and functional connectivity after transcutaneous spinal cord direct current stimulation in idiopathic restless legs syndrome.	Double-blinded, placebo-controlled	30 RLS patients and 20 matched healthy controls	Anodal tsDCS and sham. MRI and fMRI data with voxel-based morphology and resting-state functional connectivity analysis. International RLS Rating Scale (IRLS-RS) and Pittsburgh Sleep Quality Index (PSQI).	Sham treatment group: no significant change in IRLS-RS and PSQI scores after tsDCS. Anodal treatment group: significant ↓ in IRLS-RS and PSQI scores after tsDCS up to 2 weeks. Anodal treatment group: significant ↓ of gray matter volume in different brain regions. Change in functional connectivity between different brain region.
Zeng et al. (80)	Transcutaneous spinal cord direct current stimulation modulates functional activity and integration in idiopathic restless legs syndrome.	Double-blinded, placebo-controlled	30 RLS patients and 20 matched healthy controls	Anodal 2 mA tsDCS and sham. International RLS Rating Scale (IRLS-RS) and Pittsburgh Sleep Quality Index (PSQI). Resting-state fMRI data.	tsDCS improved the sleep and RLS symptoms (PSQI, IRLS-RS). Brain changes in the voxel-wise fractional amplitude of low-frequency fluctuations, regional homogeneity and weighted degree centrality are described and correlated with sleep and RLS symptom scores.

↓ = decrease.

These studies together with the studies by Heide et al. (64) and by Monte-Silva et al. (27) on long-term potentiation of the motor cortex by repetitive tDCS and by Fregni et al. (34), mentioned above, which demonstrated long-lasting positive effects of tDCS in the treatment of patients with chronic pain, show that repetitive direct current stimulation may have the potential to induce positive long-lasting therapeutic effects in various diseases (27, 34). However, in order to establish tsDCS as a treatment method for RLS, more studies are needed to provide sufficient evidence for long-term effects.

### TMS and spinal cord stimulation as a treatment alternative for RLS

In a review about TMS as a treatment option for various sleep disorders, Nardone et al. (82) present four studies that prove the effectiveness of repetitive TMS (rTMS) in RLS patients (55, 82–85). The authors summarized that both, high-frequency (HF) and low-frequency (LF) rTMS, applied over the primary motor cortex or the supplementary motor cortex, seem to have transient beneficial effects in patients with RLS. Liu et al. (85) provided evidence for the efficacy of rTMS by additional functional magnetic resonance imaging (fMRI): besides the improvement of the IRLSSG scale score after bilateral stimulation of motor area 1 (M1), patients also showed an increase in functional activity, measured as the amplitude of low-frequency fluctuations (ALFF), in the sensory-motor regions and in the occipital lobes. Lanza and colleagues (86) concluded that excitatory stimulation of M1 might inhibit the thalamic inputs. In a study, Lanza et al. (55) provided evidence that the indexes of rTMS on S1-M1 of excitation and inhibition indicate a pattern of cortical neurotransmitter imbalance mainly involving gamma-aminobutyric acid (GABA)ergic and glutamatergic circuitries, as well as dopamine levels in the dorsal striatum, which fits well with the current theory of the pathogenesis of RLS.

However, just like with tsDCS, there are still some pitfalls to consider with this treatment method (87). These range from the small sample sizes, the use of self-reported scales of sleep quality or RLS symptoms as the only outcome variable in many studies, technical difficulties in recording the motor responses from lower limb muscles in RLS patients, the reproducibility of the results and the relatively complicated application using the magnetic coil. At least the first two issues also apply to tsDCS. Although the results of TMS in RLS patients are undoubtedly promising, the limitations concerning TMS might make the use of tsDCS in everyday clinical practice a little easier.

To our knowledge, only one article about the effectiveness of invasive spinal cord stimulation (SCS) in RLS has been published so far (88). In this publication, Pagani-Estévez et al. (88) reported a series of 16 unrelated cases without a randomized, double blind controlled design, thus presenting insufficient evidence of the effectiveness of this treatment method. Further controlled studies about the effectiveness of SCS in RLS patients are urgently needed, particularly considering that neurostimulators for a wide variety of diseases (sleep-related breathing disorders or chronic pain) are becoming increasingly important.

## Conclusion

tDCS and tsDCS are established among neuromodulation methods. Like in TMS, the effects of tDCS and tsDCS depend on polarity duration and intensity of stimulation, which are important features of neuroplastic changes (25). Although, the results of the different studies presented in this review are promising, there is a lack of long-term, randomized-controlled trials in the current space. Additional research is warranted to further support the clinical use of these emerging treatment modalities in pain management, in improvement of the level of physiological functions and as a well-tolerated alternative to drug treatment in RLS patients.

## Author contributions

CD: Conceptualization, Investigation, Writing – original draft. CB: Conceptualization, Funding acquisition, Supervision, Writing – review & editing.
